# Microbiological Hazards Associated with the Use of Oligocene Waters: A Study of Water Intakes in Warsaw, Poland

**DOI:** 10.3390/microorganisms11040826

**Published:** 2023-03-24

**Authors:** Ewa Karwowska, Ewa Miaśkiewicz-Pęska, Katarzyna Gołębiewska, Paulina Tomaszewska

**Affiliations:** Department of Biology, Faculty of Building Services, Hydro and Environmental Engineering, Warsaw University of Technology, Nowowiejska 20, 00-653 Warsaw, Poland

**Keywords:** Oligocene waters, microorganisms, microbiological hazard, antibiotic resistance

## Abstract

Oligocene waters are widely recognized as excellent sources of drinking water. Due to the belief in their good quality, the water from Oligocene intakes in Warsaw, Poland, is made available to users without prior treatment or disinfection. The present study aimed at assessing possible microbiological risks associated with the use of this water. The occurrence of microbiological contaminants in selected intakes was evaluated, in addition to an assessment of possible changes in the microbiological quality of the water under typical storage conditions. The possibility of antibiotic resistance in bacteria isolated from Oligocene water samples was also investigated, as was their sensitivity to selected disinfectants. A small number of bacteria—27.0 ± 60.8 CFU/cm^3^ and 3.0 ± 3.0 CFU/cm^3^—were found in Oligocene water intakes for psychrophilic and mesophilic bacteria, respectively. Fecal bacteria were not detected. Bacteria present in Oligocene waters showed the ability to multiply intensively during standard water storage; this was especially true for mesophilic bacteria in water stored at room temperature. In some samples, bacterial counts reached 10^3^–10^4^ CFU/cm^3^ after 48 h. Almost all bacterial isolates were resistant to the commonly used antibiotics: ampicillin, vancomycin and rifampicin. The bacteria were also insensitive to some disinfectants.

## 1. Introduction

Groundwater has always been considered a valuable and safe source of drinking water [1,2]. Thanks to an effective separation from the Earth’s surface and the presence of geological layers with low permeability and increased isolation parameters, these waters are relatively well protected from the penetration of various types of contaminants of both a chemical and microbiological nature. Groundwater naturally contains a certain small number of microorganisms. These are mostly microorganisms responsible for the processes of decomposition of organic matter and the processes of oxidation and reduction of the mineral substances contained in water (ferrous and manganese bacteria and nitrifying, denitrifying and sulfate-reducing bacteria, among others). The number of bacteria decreases significantly with increasing depth, although they can be detected sporadically even at depths of up to 2500 m [3].

The natural microflora of groundwater consists mainly of microorganisms that are well adapted to oligotrophic, nutrient-poor conditions. Microbial communities in groundwater are usually characterized by a limited biodiversity and relatively stable species composition. As shown, representatives of diverse heterotrophic *Proteobacteria*, *Actinobacteria*, *Firmicutes* and *Bacteroidetes* dominate. Bacteria belonging to *Acidobacteria*, *Chloroflexi*, *Verrucomicrobia* and *Nitrospirae* were also detected in groundwater [4].

The microflora of groundwater can include both autochthonous microorganisms, which can reach an abundance as high as 10^3^/cm^3^, and allochthonous microflora, which enter water along with contaminants, mainly due to the discontinuity of the layer isolating the aquifer from the ground surface or the horizontal migration of contaminants in the ground. The allochthonous microflora show a limited survival time in deep waters, although a periodic increase in the abundance of this type of microorganism is possible within the first 1–7 days after the occurrence of contamination [5]. It has been observed that some bacteria characteristic of surface water can also occur in groundwater and even constitute the dominant microflora in it [6].

In recent years, studies have indicated that as a result of anthropogenic impact, groundwater resources around the world are increasingly vulnerable to contamination; therefore, their quality should be meticulously monitored [7]. Despite the general belief in the relative sanitary safety of groundwater, especially deep water, numerous examples of waterborne disease outbreaks linked to contaminated groundwater reservoirs and intakes have been reported worldwide [8,9]. Studies in Scandinavian countries have suggested that up to more than 70% of outbreaks may be considered, while studies in the US have shown that 52% of waterborne disease outbreaks are linked precisely to poor groundwater quality [8].

The basic range of microbiological tests used for drinking water, including routine analyses of the presence of fecal indicator bacteria in drinking water, may not provide a complete picture of the risks associated with the presence of potentially hazardous microorganisms in water and their possible proliferation under conditions of typical intake operation [8]. While in the case of large-scale water supply systems meticulous monitoring is a matter of course, estimating the possible level of danger on the basis of the available amount of data is often very difficult in the case of small, individual water intakes.A problem that has attracted the attention of many researchers in recent years is the possibility of the presence of antibiotic-resistant bacteria in groundwater, including potentially pathogenic microorganisms. There are also reports that drinking water distribution systems may be one of the sites for the spread of drug-resistant traits in microbial communities [10].

The groundwater occurring in Oligocene formations in Warsaw and the surrounding area is one of the most valuable groundwater reservoirs in Poland. The artesian or subartesian aquifer is associated with glauconitic fine- and medium-grained sands of the Lower Oligocene, occurring at a depth of 200–260 m. It covers a region called the Warsaw Basin, with an area of 14,928 km^2^ and disposable resources estimated at 372,146 m^3^/d [11]. A characteristic feature of the Oligocene deposits is their variable thickness, which ranges from a few to approximately 80 m (mostly 60–80 m), 75% of which are aquifers [12]. Paleohydrogeological studies have shown that the Oligocene formations in the area of Warsaw are 2000–70,000 years old [11].

Warsaw Oligocene water is moderately hard or soft, with a slightly alkaline pH (the pH of the water is 6.9–7.52). It exhibits a stable hydrochemical composition of the four-ion type, HCO_3_-Cl-Na-Ca, and mineralization at the level of 542.5–640 mg/dm^3^ [13]. The waters are completely odorless and have a good taste. They contain 50–120 mg/dm^3^ NaCl and 60 to 80 mg/dm^3^ Ca (HCO_3_)_2_ [14].

Currently, there are 107 publicly accessible Oligocene wells in Warsaw. They are located in different districts of the city and on both sides of the Vistula River. The typical depth of Oligocene wells is 220–270 m, while their capacity is at the level of 30–50 m^3^/h [12]. The maximum possible intake of Oligocene water from wells in Warsaw, estimated at 20,000 m^3^/d, does not make a quantitatively significant contribution to the city’s overall water supply. Nevertheless, it can be treated as a valuable strategic reserve in emergency situations [15].

The water from Oligocene intakes in the Warsaw area is considered free of both chemical and biological contaminants; hence, it is permitted for consumption without prior treatment and disinfection. Routine water monitoring tests are carried out by the district sanitary–epidemiological station at a frequency of once every two months (if the results do not comply with sanitary requirements, the frequency of tests is increased). The sanitary–epidemiological station does not perform review monitoring in Oligocene water intakes. Conducting such tests is at the discretion of the owner of the intake, and their frequency is determined individually for each intake.

Oligocene waters in Warsaw are widely recognized as an excellent source of drinking water. There is a belief among its users that it is of better quality than water from the municipal water supply [16]. It is often consumed directly from the tap, especially in the summer months, by children and adolescents and people who participate in sports. Many people also take Oligocene water for domestic use, usually using reusable plastic containers and storing the water at home for up to several days.

Despite their widespread use, Oligocene waters, including those from Warsaw intakes, have never been the subject of more extensive studies to assess whether they are indeed fully microbiologically safe and whether the way they are used somehow implies sanitary safety. The present study assesses the occurrence of potential contaminants of a microbiological nature in Oligocene water taken from randomly selected intakes from the Warsaw area. It also as attempts to estimate possible changes in the microbiological quality of the water under conditions of typical storage. In addition, attention was paid to the possibility of antibiotic-resistant bacteria in Oligocene water and their resistance to selected disinfectants.

## 2. Materials and Methods

**Figure 1:**
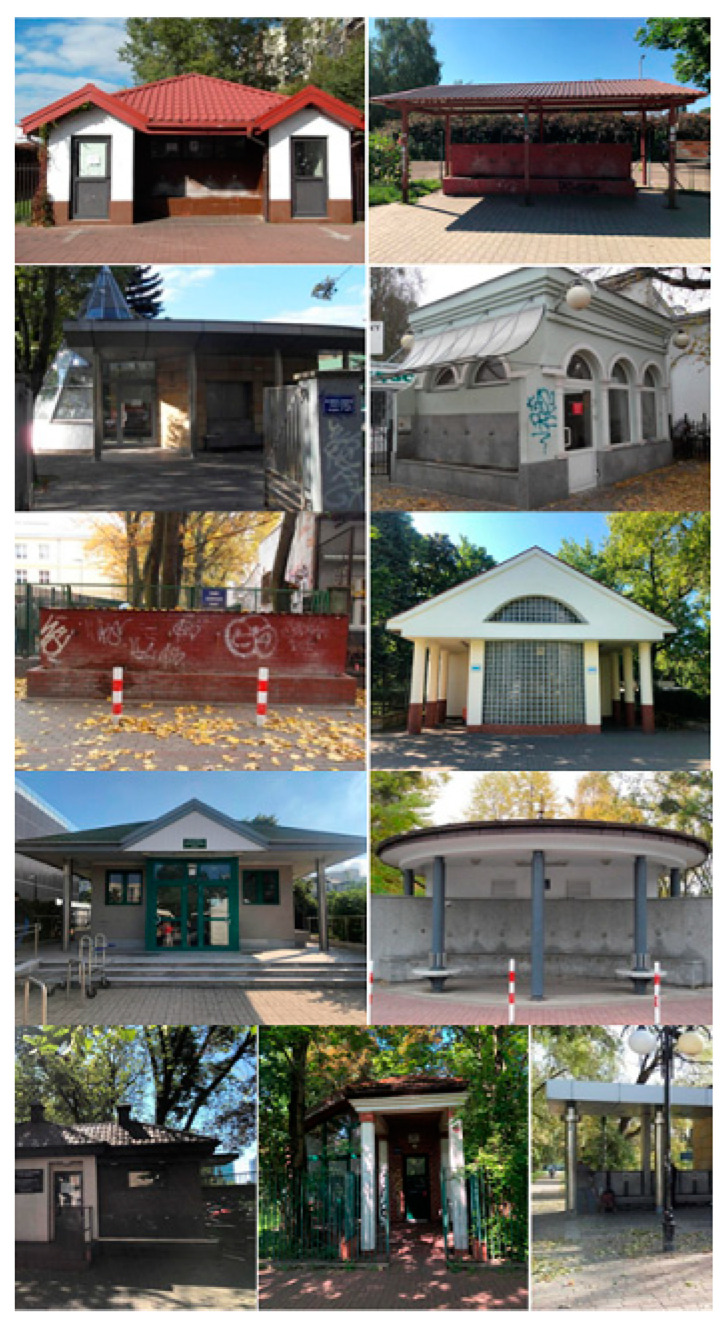
Oligocene water intakes included in the study.

Warsaw Oligocene water intakes are mostly located in brick buildings, which provide external intakes in the form of faucets on the outer wall of the building, and are not operated for most of the year.Internal intakes—used in the winter—are located inside the building to protect the water from the harmful effects of frost.Some of the Oligocene water intakes are fenced and open only during designated hours. Some of them are equipped with monitoring systems. Most of the intakes are located in residential areas. A total of 11 Oligocene water intakes, located in different parts of the city on both sides of the Vistula River, were selected for this study (Figure 1). The depth of the wells included in the study ranged from 210 to 262 m.

Before each sample was taken, the water was discharged in a calm stream for 3 min, and the end of the spout was then disinfected in a flame. Water samples were taken into sterile glass bottles with a lapped stopper and a capacity of 300 cm^3^ and into 0.5 dm^3^ plastic bottles of bottled mineral water.Two water samples were taken from each intake in parallel. The samples were transported to the laboratory within 2–3 h, depending on the location of the intakes, in cooling conditions. After the samples were delivered to the laboratory, microbiological quantitative testing of the samples was carried out the same day over the next few hours [17].

The microbiological study of Oligocene waters carried out in this research to assess the potential microbiological risks associated with their use was carried out in two stages. During the first step, the focus was on quantitative studies of the water samples taken from randomly selected intakes, taking into account both the total number of psychrophilic and mesophilic bacteria and indicators of fecal pollution (*Escherichia coli* and enterococci). An attempt was then made to estimate the potential for microbial multiplication in water under different storage conditions. The second stage of the study focused on the phenomena associated with the occurrence of bacteria in Oligocene waters that are resistant to selected antibiotics and demonstrate resistance to selected disinfectants.

In the first stage of the study, water samples were taken from six randomly selected Oligocene intakes located in different parts of Warsaw (three on each side of the Vistula River).

Determinations of *E. coli* and fecal enterococci in water were carried out using the filtration method. *E. coli* was cultivated on an agar medium with triphenyl-tetrazolium chloride (TTC) and tergitol (Biomaxima S.A., Lublin, Poland). The cultivation medium, according to Slanetz and Bartley (BTL Ltd., Łódź, Poland), was applied for fecal enterococci. A volume of 100 cm^3^ of tested water was passed through a membrane filter with a pore diameter of 0.45 µm. The filter was then placed on the surface of the corresponding medium and incubated for 48 h at 37 °C.

In order to confirm the presence of *E. coli* in the water, the material taken from the yellow-colored colonies obtained on the surface of the filter was transferred to lactose brilliant green bile broth (Merck Polska, Warsaw Poland) and peptone broth with tryptophan (Merck) (medium for detecting the indole-producing bacteria). The cultures were then incubated for 24 h at 44 °C. The presence of gas, indicating the occurrence of a lactose fermentation reaction on the brilliant green bile broth and the presence of indole formed from tryptophan with the participation of bacteria, wastaken as a positive result. The results for coliforms and fecal enterococci were reported as the number of CFU/100 cm^3^ of water.

A determination of the number of mesophilic and psychrophilic bacteria was performed with Koch’s growth method [18], using the pour plate technique on a nutrient agar medium (Biocorp Poland Ltd., Warsaw, Poland). Analyses of both the undiluted samples and samples after their serial dilution in the range of 10^−1^–10^−3^ were performed. The cultures were prepared in two parallel replicates. Mesophilic bacteria were incubated at 37 °C for 48 h, while psychrophilic bacteria were incubated at 20 °C for 72 h. The results of the bacterial counts were reported as the number of colony-forming units (CFU) in 1 cm^3^ of water.

The study of the process of multiplication of the microorganisms present in the water within 48 h after collection from the intake was carried out by storing water samples from each intake at 4–7 °C and 20–22 °C, respectively, for 48 h. After this time, all the microbiological tests were repeated.

In the second stage of the research, water sampling was carried out from five randomly selected Oligocene water intakes. Due to the low number of bacteria found in the Oligocene water during the first stage of the study, the filtration method, using membrane filters as in stage I, was used to obtain the number of bacterial colonies sufficient for further study. Water samples of 10 cm^3^, 50 cm^3^ and 100 cm^3^ were filtered for each tested intake. The filters were then placed on Tripticasein Soy Lab Agar (Biomaxima S.A.) medium and incubated at 37 °C for 24 h. After this time, the number of colony-forming units per 100 cm^3^ of water was determined based on the number of colonies that appeared on the filters. On the basis of culture and morphological characteristics (Gram-staining method), different bacterial strains were selected from all the tested samples. Pure strains for further studies were obtained by the successive reduction of cultures on TSA medium, using the streak plate method. The cultures were incubated for 24 h at 37 °C each time. The choice of incubation temperature was dictated by the focus of further research on bacterial microflora capable of growing at human body temperature and are thus able to pose a potential health risk. A total of 25 different bacterial isolates were obtained from all water intakes. These were then tested for resistance to selected antibiotics and disinfectants.

To determine the sensitivity of bacteria to antibiotics, antibiograms were performed using the disk-diffusion method (Kirby–Bauer method). A surface culture of a dense suspension on Mueller–Hinton medium was used to obtain uniform bacterial growth. Discs with the antibiotics: vancomycin, ampicillin, rifampicin and gentamicin (Emapol Ltd., Gdańsk, Poland) were placed on the surface of the culture thus prepared. The samples were then incubated at 37 °C for 24 h. To estimate the sensitivity of bacteria to a given antibiotic, the diameter of the zone of bacterial inhibition was measured. The results were interpreted according to the European Committee on Antimicrobial Susceptibility Testing Breakpoint tables for interpretation of MICs and zone diameters [19].

In order to estimate the susceptibility of the bacteria to the selected disinfectants, a surface culture of a dense suspension of each strain was performed on TSA agar medium.Sterile disks, which were previously soaked in the following agents, were placed on the surface of the culture: 1% aqueous solution of Cl_2_ (“chlorine water”); 80% ethyl alcohol; 1% aqueous solution of silver nitrate. The samples prepared in this way were incubated at 37 °C for 24 h. After this time, observations were made of the zones of inhibition of bacterial growth around the disc with the given disinfectant.

## 3. Results

The quantitative testing of water samples carried out showed that all the analyzed Oligocene water samples were free of microorganisms indicative of fecal contamination. At both the time of collection and after a storage (regardless of conditions), no fecal enterococci were detected in the water from the studied Oligocene intakes. The confirmatory test, which was carried out for “doubtful” colonies obtained on *E. coli* culture medium, also provided a negative result. This allows us to conclude that the tested Oligocene water in all cases met the basic sanitary requirements stipulated by law for water intended for drinking purposes.

**Figure 2:**
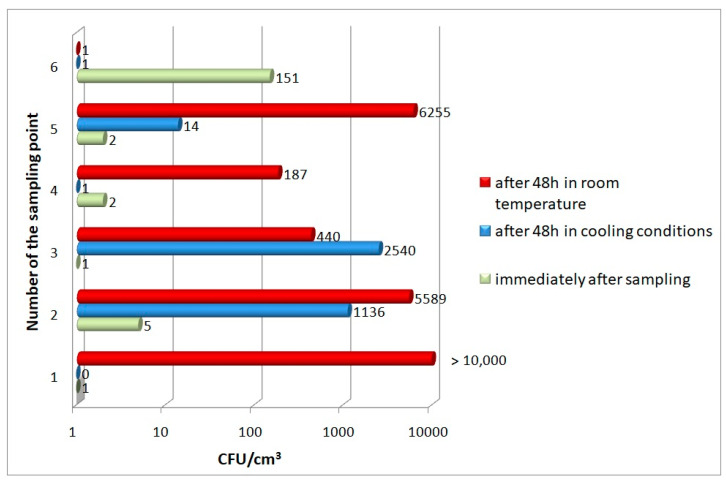
The numbers of psychrophilic bacteria in Oligocene water samples.

In the quantitative analysis of psychrophilic and mesophilic bacteria present in samples of the tested Oligocene water, only few bacteria were found. The number of bacteria growing at 20 °C (psychrophilic) was 27.0 ± 60.8 CFU/cm^3^, while the number of bacteria growing at 37 °C (mesophilic) was 3.0 ± 3.0 CFU/cm^3^. In samples taken from one of the intakes, the content of psychrophilic bacteria was found to be higher than in the others—76 and 226 CFU/cm^3^ (respectively, in the samples taken in parallel).The research to trace the process of bacterial proliferation in Oligocene water under different storage conditions showed that in the case of water samples from some of the intakes, there was an unusually intensive proliferation of psychrophilic bacteria. In comparison with the baseline value, the number of these bacteria increased in one case by more than 200 times, while the number increased by more than 2500 times in another case. Even more disturbing results were obtained for samples of Oligocene water stored at room temperature (about 20–22 °C), conditions very similar to the way in which users usually keep Oligocene water in their homes. It was found that after 48 h of storage, the number of psychrophilic bacteria increased for five of the six samples tested, with the number reaching several thousand colony-forming units in 1 cm^3^ of water for intakes 1, 2 and 5 (for sample 1, it even exceeded 10,000 CFU/cm^3^) (Figure 2).

**Figure 3:**
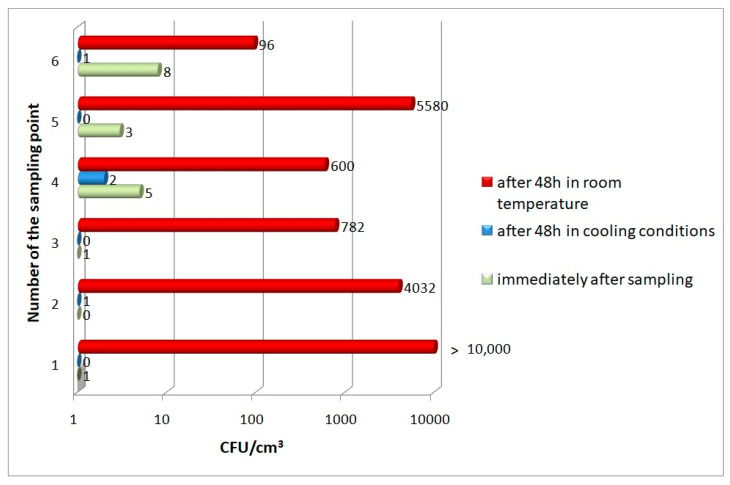
The numbers of mesophilic bacteria in Oligocene water samples.

The determination of the number of mesophilic bacteria in water samples stored under different conditions showed that in most cases, storing water for 48 h under refrigeration (4–7 °C) did not cause a significant increase in the number of these bacteria. Moreover, in some samples, mesophilic bacteria were not detected after 48 h, despite their previous presence in the water at the time of intake. The situation was completely different for samples stored at room temperature. An unusually intense proliferation of bacteria capable of growing at 37 °C was observed in water from all six intakes studied, despite the fact that the temperature at which they were stored was lower than the optimum temperature for them. Bacterial numbers increased from several hundred to more than 10,000 times, depending on the sample (Figure 3).

Tests conducted in the second stage of the work again showed that the number of bacteria present in samples of Oligocene water from the selected sources directly at the time of collection was not very high: at the level of 2.3 ± 2.2 CFU/cm^3^. By using the filtration method, it was possible to obtain an adequate number of colonies for further studies. The analysis of the morphology of the isolated bacteria showed that Gram-positive bacteria predominated in the Oligocene waters (22 out of 25 isolates). More than half of the isolates were Gram-positive, spore-forming bacilli; 28% were spherical forms (mainly staphylococci); and 12% were Gram-negative bacteria. One *Corynebacterium*-like microorganism was also isolated.

**Figure 4:**
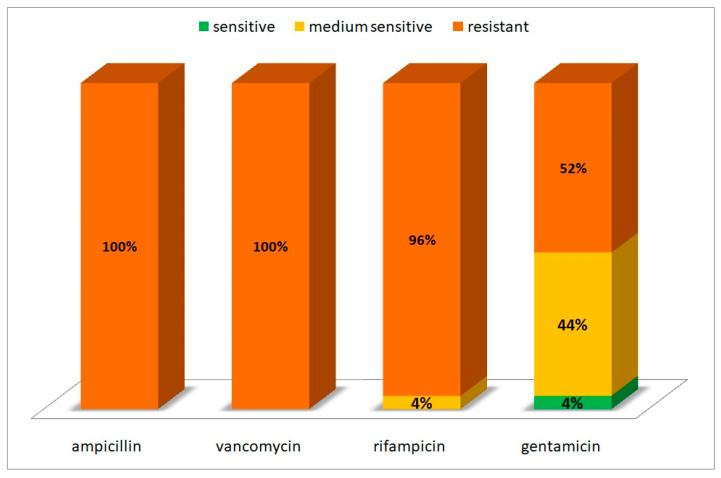
Antibiotic resistance of bacteria isolated from the samples of Oligocene waters.

Resistance tests on isolates obtained from the Oligocene water samples yielded a surprising yet highly important result. It was demonstrated that all isolated bacterial strains were characterized by resistance to ampicillin and vancomycin. Rifampicin inhibited the growth of only one of the strains tested; therefore, it can be concluded that the microflora from the intakes included in the study showed far-reaching resistance to this antibiotic as well. Resistance to the aforementioned antibiotics occurred equally in both Gram-positive and Gram-negative bacteria. The only antibiotic that demonstrated activity against at least some isolates was gentamicin (Figure 4). One of the isolates was found to be sensitive to gentamicin, while a further 11 strains were intermediately susceptible. Antibiotic resistance tests showed that bacterial resistance to ampicilin and vancomycin was not directly related to cell morphology. A Gram-positive staphylococcus was the only isolate to demonstrate a moderate sensitivity to rifampicin; the remaining isolates, both Gram-positive and Gram-negative, were resistant to this antibiotic. In the case of gentamicin, it was found that the moderately sensitive strains included mainly Gram-positive bacteria—bacilli and staphylococci. The only strain classified as susceptible was also Gram-positive bacillus.

A parallel study of the effectiveness of selected disinfectants on bacteria isolated from the Oligocene water intakes showed that all 25 strains were resistant to both chlorine (in the form of 1% chlorinated water) and 80% ethanol. Weak growth inhibition was noted only when a 1% silver nitrate solution was used as a disinfectant.

## 4. Discussion

The sanitary safety of drinking water, including groundwater, is a fundamental issue from the perspective of protecting the health of its potential users. Although groundwater is usually well protected from external contamination due to its continuous and impermeable soil layer, the possibility of the microbiological contamination of groundwater reservoirs located in areas of surface water influence must be taken into account, in addition to the ingress of contaminants found in the ground into groundwater.

As confirmed by numerous scientific research results, the microbiological contamination of groundwater is an increasingly critical problem from the perspective of ensuring sanitary safety [1,20]. Numerous studies confirmed the presence of pathogenic or potentially pathogenic microorganisms in groundwater [1,21,22] (among others, bacteria such as: *Arcobacter butzleri*, *Campylobacter* spp., *E. coli*, *Helicobacter pylori*, *Legionella* spp., *Salmonella* spp., *Shigella* spp., *Vibrio cholerae* and *Yersinia* spp. and protozoa such as *Cryptosporidium* spp., *Encephalitozoon intestinalis*, *Giardia lamblia* and *Naegleria fowleri*) [2,23]. Powell et al. [1] reported the presence of enteric viruses in deep groundwater in urban consolidated sandstone aquifers in the UK. Protozoa are usually detected in aquifers closer to the surface, but it should not be ignored that protozoa of the genus *Cryptosporidium* have also been detected in intakes whose design prevented the direct penetration of contaminants from the surface [24]. Bacteria and viruses typical of municipal wastewater were detected up to a depth of 90 m below ground level [25]. Microorganisms indicative of groundwater contamination by wastewater were detected, among others, in sandstone aquifers underlying the cities of Birmingham and Nottingham, England [25]. The vulnerability of groundwater to contamination depends, among other factors, on parameters such as the depth of the groundwater table, the degree of isolation of groundwater from the land surface, the sorption properties of adjacent soils and the proximity to the source of contamination [26].

Microorganisms have been detected in water from various depths. Haveman and Pedersen [27] isolated abundant bacteria in groundwater from Fennoscandian Shield sites in Finland and Sweden from depths ranging from 65 to nearly 1400 m, while their abundance reached as high as 3.7 × 10^5^ cells/cm^3^.

In their study on groundwater from Olkiluoto, Finland, Pedersen et al. [28] found that bacterial abundance in water samples taken from deep boreholes from depths of 35–742 m reached 5.7 × 10^4^ cells/cm^3^. Bacterial abundance remained at a similar level until a depth of approximately 250 m, while even a slight increase was recorded at a depth of approximately 300 m. Against the background of the above data, it can be concluded that the total number of bacteria in the samples of Warsaw Oligocene water is comparatively low, as even in samples with the highest content it did not exceed the level of 300 CFU/cm^3^. This is probably facilitated by the relatively good separation of the Oligocene aquifer as well as the depth of the wells supplying water to the studied intakes.

The susceptibility of groundwater to contamination depends on many different factors. One of the most important factors is the depth of the groundwater table, which affects the speed of contaminant movement [29]. In porous formations, groundwater can move at speeds ranging from fractions of a millimeter per day to tens of meters.In heavily sealed and karstic rocks, the movement of groundwater is rapid; its speed can reach up to several hundred meters per day. This results in poor filtration of the water and thus the penetration of more contaminants into its depths. Natural filtration processes occurring in the soil can be an effective factor in eliminating microbial contaminants coming from the surface. They can also contribute to the inactivation of potentially pathogenic microorganisms and thus reduce the risk of infection [8,24]. Limiting factors for the presence of microorganisms at great depths are the limited availability of oxygen, the lack of water-filled fractures that can provide a habitat for microorganisms, the lack of nutrient substrates and, in the case of very deep deposits, elevated temperatures [28,30].

Oligocene waters from the Warsaw Basin area are relatively well-isolated from their surroundings.Thus, their susceptibility to microbiological contamination is not very high. Thanks to this good natural isolation, which is formed by the more than 100 m thick layer of Pliocene clay pack above, the Oligocene aquifer in the Warsaw area has a very low risk of surface pollution [11,12]. The isolation layer below the Oligocene formations consists of very poorly fractured marls of the Upper Cretaceous [31]. A possible threat in terms of water quality may be geogenic pollution, which manifests itself as increased water color associated with the infiltration of water from the Miocene horizon and chloride content in the western part of the Warsaw Basin. Elevated concentrations of manganese, iron and ammonia are recorded in places. These compounds can be easily removed by water treatment processes [11,32].

In the present study, bacteria associated with fecal pollution were found to be absent from the water. Similarly, Yahaya et al. [33] showed that borehole groundwater samples did not contain coliform bacteria, although the total bacterial count was in the range of 140 to 12,000 CFU/cm^3^. Coliform bacteria are considered a good indicator of the fecal contamination of groundwater, among other things, due to their stated ability to move in the ground environment [21]. However, it should be taken into account that the failure to find typical fecal bacteria in water is not a conclusive criterion for sanitary safety, as it has been observed that sometimes the presence of potentially pathogenic viruses can be indicative of fecal contamination of groundwater while typical bacterial indicators of fecal contamination such as *E. coli* and fecal enterococci are not detected [25].

The Oligocene waters investigated in this study are not subjected to treatment or disinfection processes; therefore, a number of recommendations of a sanitary nature have been made regarding their use. According to the guidelines for the use of water from Oligocene intakes, the water should be stored at 2–8 °C and preferably consumed within 24 h and within a maximum of 4 days. Containers into which Oligocene water is taken should be properly cleaned before each subsequent intake and should not come into contact with sunlight while there is water stored in them.

Despite the recommendations of the State Sanitary Inspectorate that Oligocene water should be used within 24 h of extraction from the intake, it is sometimes stored even for several days in practice. The results of a survey on the use of deep-sea water in the households of Warsaw residents conducted by Parzuchowska and Ostalska [16] showed that only less than 11% of users of Oligocene intakes declared that they consumed water within a few hours of taking it. Of these users, 52.50% admitted that they stored the water for 1–2 days, while approximately 31% stored it for 3–7 days. Nearly 6% of users stored water for more than a week. Water is most often kept in plastic containers (93.5% of users), and more than 43% of them are open containers. Only just over 25% of respondents said they used detergents to periodically wash their containers. The water is most often left at room temperature (87%). A refrigerator (13%), garage/basement (15%) or balcony (27%) were cited as other storage locations. The cited data demonstrate that the actual behavior of residents deviates from the recommendations of the State Sanitary Inspectorate.

As this study has shown, the storage of water at room temperature can result in notable microbiological hazards, with the most significant phenomenon appearing to be the proliferation of mesophilic bacteria in the water. This is an extremely important observation in light of the fact that it is among mesophilic bacteria that potentially pathogenic species or strains can be found.The safe consumption of such water requires heat treatment, with the understanding that brief boiling may not eliminate all microorganisms present in the water. Alarmingly, some of the microorganisms present in the water can also multiply under refrigerating conditions. It is also important to emphasize the fact that despite the similar microbiological parameters of Oligocene water from different intakes at the time of sampling, its properties after a period of storage can differ greatly from each other, affecting the safety of its consumption. The results obtained suggest the possibility of an increase in microbial contamination of Oligocene water even if it is stored in the manner recommended in the sanitary guidelines.

Studies conducted in recent years confirmed that the phenomenon of bacterial resistance to antibiotics and the process of genes that determine drug resistance spreading in the environment increasingly affect groundwater [34]. Numerous studies show that a horizontal gene transfer is possible in groundwater, allowing microorganisms to acquire new properties, including a resistance to changing environmental conditions and the presence of a specific type of contaminant [4]. Li et al. [21] showed that *E. coli* and *Enterococcus* sp. bacteria detected in groundwater were characterized by resistance to at least one antibiotic, with 63.6% of *E. coli* isolates and 86.1% of *Enterococcus* sp. showing multi-resistance towards three or more antibiotics. The tested isolates of both species were most often resistant to antibiotics such as tetracycline and chloramphenicol. In addition, *E. coli* isolates showed a resistance to trimethoprim/sulfamethoxazole and azithromycin, while enterococci were resistant to tigecycline, quinupristin/dalfopristin, linezolid, erythromycin and ciprofloxacin. A study of groundwater conducted in Kenya by Wahome et al. [35] showed that pathogenic and potentially pathogenic bacteria isolated from water samples (including those of the genera *Salmonella*, *Shigella*, *Vibrio*, *Escherichia* and *Pseudomonas*) demonstrated a high resistance to sulphamethaxazole, kanamycin and ampicillin. All *Salmonella* sp. isolates were resistant to ampicillin and kanamycin, while some were also resistant to gentamicin and cotrimoxazole. A total of 50% of *Shigella* sp. isolates were resistant to streptomycin. In the case of *E. coli*, all isolates were characterized by resistance to ampicillin, tetracycline, cotrimoxazole, streptomycin and sulphamethaxazole, while half of them were also resistant to gentamicin and chloramphenicol. Additionally, all *Pseudomonas* sp. strains isolated from groundwater showed multiresistance to antibiotics such as ampicillin, cotrimoxazole, streptomycin, kanamycin and gentamycin.

Szekeres et al. [7] confirmed the prevalence of drug resistance genes in the groundwater environment. They detected a correlation between groundwater contamination and the presence of antibiotic resistance genes in groundwater, mainly those such as tetC, tetO and tetW. Gowrisankar et al. [36] isolated pathogenic antibiotic-resistant bacteria from groundwater that came into contact with surface water in post-flood areas in India. They showed resistance to antibiotics such as ceftriaxone, doxycycline and nalidixicacid, but were sensitive to chloramphenicol, tetracycline, ciprofloxacin and norfloxacin.Tan et al. [37] provided numerous examples of work in which antibiotic-resistant bacteria and drug resistance determinant genes were isolated from drinking water intakes. Su et al. [10] cited some studies confirming that resistance genes to antibiotics such as sulfonamide, tetracycline, cephalosporin, chloramphenicol, and penicillin were detected in drinking water sources.

The research carried out within the present study provided very disturbing data. It turns out that despite their low abundance, bacteria isolated from samples of the Oligocene water from intakes in Warsaw could pose some kind of hazard. All isolates showed resistance to commonly used antibiotics, including those considered broad-spectrum antibiotics.Only gentamicin showed activity against some of the isolates. It should be noted that only the results obtained for gentamicin corresponded with the activity profile of the antibiotic (against Gram-positive bacteria). The widespread resistance of bacteria isolated from Oligocene water samples to the broad-spectrum antibiotics ampicillin and rifampicin was surprising, as was the fact that vancomycin, dedicated against Gram-positive bacteria, had no effect on Gram-positive bacilli and staphylococci.

The fact that the drug-resistant strains came from intakes located at great distances from each other is noteworthy. This may indicate the very wide range of the observed phenomenon. The common resistance of all the bacteria isolated from Oligocene intakes can be related to the horizontal transfer of respective genes. The transfer of Van-group genes determining the resistance to glycopeptide antibiotics, including vancomycin, was previously described in terms of a gene transfer from enterococci to staphylococci [38]. Non-inherited bacterial resistance related to natural insensivity, temporary inhibition of replication or biofilm formation should be also considered as a potential mechanism of microbial resistance [39]. In view of the above, it should not be ignored that drug-resistant microorganisms entering the human gastrointestinal tract with ingested water can transmit antibiotic resistance determinants to intestinal bacteria and consequently contribute to the further spread of drug-resistant traits in the environment [7]. Moreover, the phenomenon of drug resistance was accompanied by resistance of the isolated bacteria to common disinfectants, including chlorine. There are littoral data confirming the co-occurrence of these phenomena. Hu et al. [34] found that one of the bacteria isolated from a drinking water source belonging to the *Pseudomonas aeruginosa* species showed resistance to antibiotics at concentrations of several hundred milligrams per liter and low concentrations of chlorine. Jia et al. [40] found up to 151 antibiotic resistance genes, belonging to 15 different types, in drinking water. They also noted that the chlorination process increased the frequency of these genes, though it caused a decrease in their diversity. The systems of genes determining multi-drug resistance were detected primarily with chlorine-resistant strains of bacteria from the genera *Pseudomonas* and *Acidovorax.*

## 5. Conclusions

The results obtained in the present study provided important information on the key risks associated with the use of water from Oligocene intakes located in Warsaw for food purposes. It was shown that at the time of intake, the water does not contain too many microorganisms; however, a worrying feature found in all dominant strains is their resistance to commonly used antibiotics, including broad-spectrum pharmaceuticals. Moreover, the drug-resistant bacteria are also insensitive to some disinfectants. Among the microorganisms in the water are both bacteria from the environment and mesophilic bacteria capable of growing at human body temperature. These bacteria show the ability to multiply intensively under the typical conditions of storage by users of water from Oligocene intakes, which can pose a real threat from the perspective of spreading drug resistance.

## Data Availability

Not applicable.
